# Shisha smoking as a possible cause of bilateral granulomatous lung lesions

**DOI:** 10.1002/rcr2.374

**Published:** 2018-10-09

**Authors:** Eun Ho Choe, Luke Sutherland, Christopher Hills, Jai‐deep Sood

**Affiliations:** ^1^ Department of Respiratory Medicine North Shore Hospital Auckland New Zealand; ^2^ Department of Medicine North Shore Hospital Auckland New Zealand; ^3^ Department of Pathology North Shore Hospital Auckland New Zealand

**Keywords:** Granuloma, lung, shisha, water pipe

## Abstract

A 19‐year‐old male who regularly smoked tobacco shisha pipes presented with pleuritic chest pain, dyspnoea, and cough. He was found to have multiple bilateral lung nodules on computed tomography. A biopsy of the lung revealed necrotizing granulomatous inflammation but without evidence of infection, foreign body, vasculitis, or malignancy. There was spontaneous and complete clinical and radiographic resolution over the next 12 weeks following cessation of shisha use.

## Introduction

Shisha smoking is an activity that is gaining popularity. Data on the harmful effects of shisha smoking are relatively scarce. We report the case of a 19‐year‐old male presenting with bilateral multiple granulomatous lung lesions likely related to shisha smoking, with complete clinical and radiological resolution following cessation of shisha use. The literature on other reported cases of pulmonary complications following shisha use has been reviewed.

## Case Report

A 19‐year‐old male presented to the emergency department with a two‐week history of pleuritic chest pain, dyspnoea, and non‐productive cough. He denied fevers, night sweats, or weight loss. He had no articular, cutaneous, or ocular symptoms. He had mild childhood asthma in the past. He was not on any regular medications. There was no significant family history. He had moved to New Zealand from Fiji eight years earlier. He had regularly smoked tobacco through a “shisha” pipe for the preceding 3 months.

On examination, he was afebrile, with a heart rate of 90 beats per minute, blood pressure of 110/80 mmHg, and oxygen saturations of 98% on air. His cardiac and respiratory examination was normal. His abdomen was non‐tender without evidence of masses. He had no peripheral lymphadenopathy. His testicular examination was normal.

A full blood count showed normal haemoglobin of 147 g/L (normal range 130–175), white blood cell count of 8.8 × 10^9^ (normal range 4–11), and eosinophil count of 0.2 × 10^9^ (normal range 0–0.5). C‐reactive protein was 25 mg/L (normal range 0–5).

**Figure 1:**
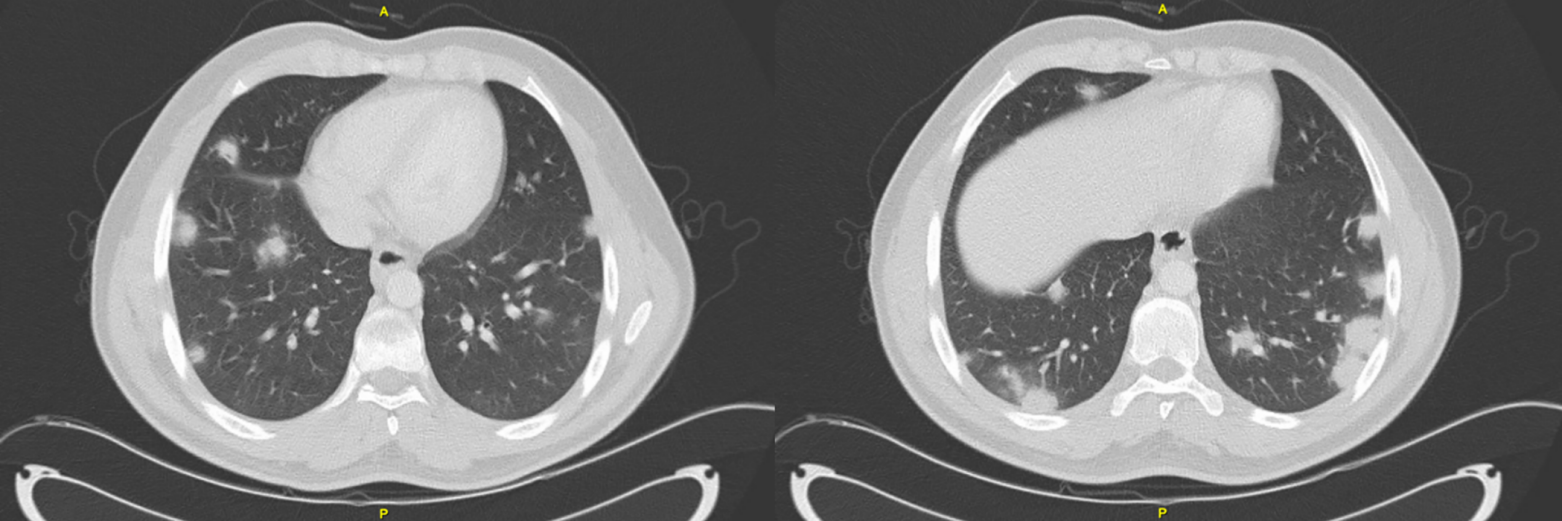
Contrast‐enhanced CT chest showing multiple poorly marginated, irregular contoured, enhancing nodules throughout both lungs.

His chest radiograph showed multiple ill‐defined opacities in both lower lung fields. A subsequent computed tomography (CT) scan of the chest and abdomen (Fig. 1) showed multiple poorly marginated and irregularly contoured enhancing nodules through both upper and lower lobes bilaterally, more numerous at the bases. There were no pleural effusions or lymphadenopathy, and appearances of the abdomen were normal.

Further blood tests showed a negative antinuclear antibody (ANA), extractable nuclear antigen (ENA) panel, anti‐neutrophil cytoplasmic antibodies (ANCA), serum angiotensin‐converting enzyme (ACE), alpha‐feto protein, Beta human chorionic gonadotropin (hCG), and Quantiferon‐Gold.

**Figure 2:**
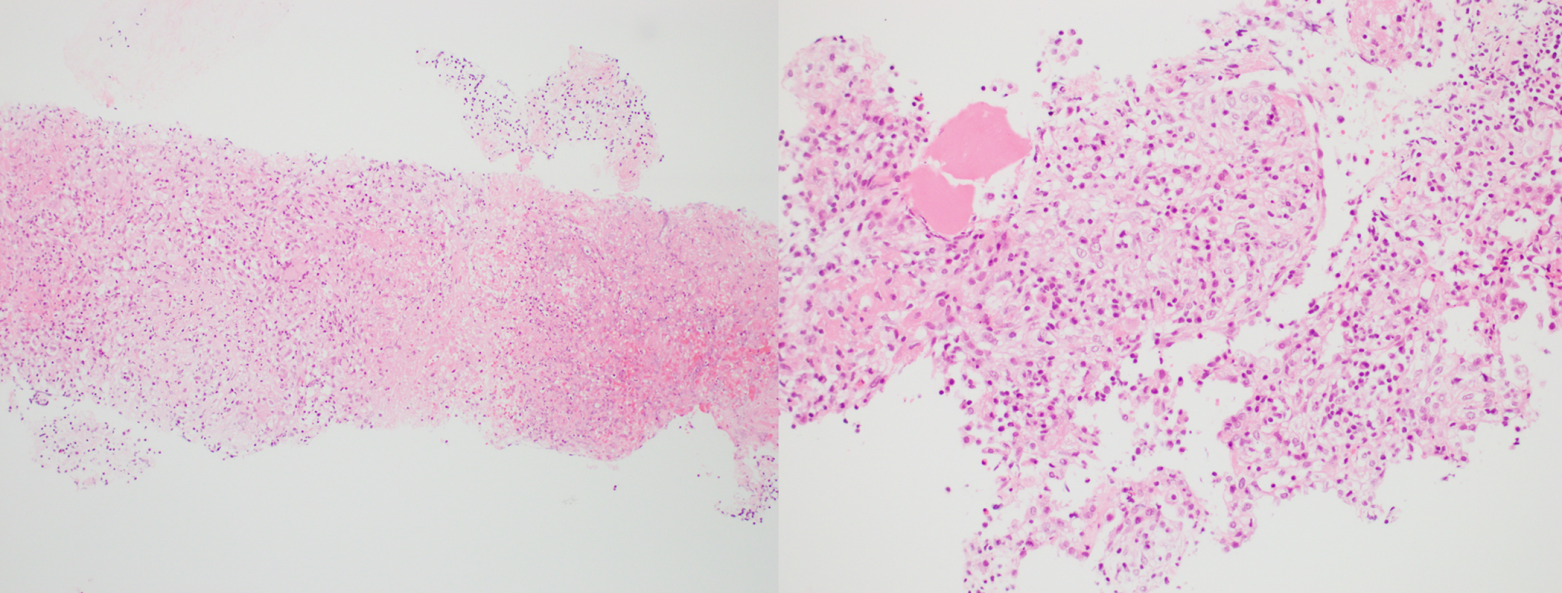
Left – low‐power view (10×) of lung biopsy showing granulomatous inflammation and necrosis. Right ‐ high‐power view (40×) showing clusters of histiocytes within alveoli.

He proceeded to a CT‐guided fine needle aspirate and core biopsy of the most peripheral lesion in the left lower lobe. Histology (Fig. 2) demonstrated preserved lung alveoli but prominent expansion of the lung interstitium with frequent necrotizing granulomas. There was a mixed inflammatory infiltrate of lymphocytes and occasional plasma cells and eosinophils. Fungal and mycobacterial stains were negative, as was culture for bacteria, mycobacteria, and fungi. Mycobacterium tuberculosis polymerase chain reaction (TB PCR) on biopsy specimen was negative. There was no evidence of malignancy, vasculitis, or foreign bodies, nor any features of Langerhans cell histiocytosis (LCH). Cell marker studies showed no evidence of monoclonality. Overall, the pathology impression was of necrotizing granulomatous inflammation.

**Figure 3:**
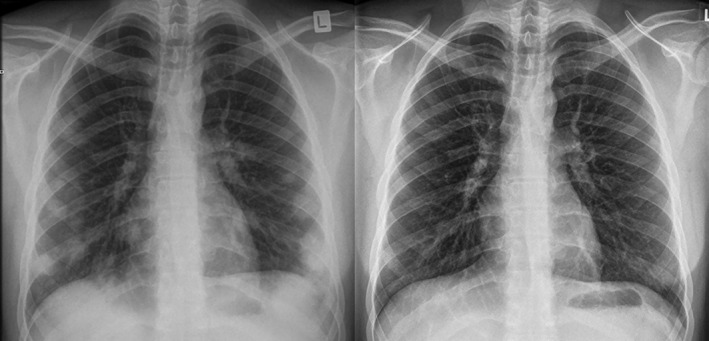
Left – chest radiograph at 2 weeks. Right – chest radiograph at 12 weeks demonstrated resolution.

The patient was given no specific treatment other than simple analgesia. While awaiting the histology results, he was discharged home with advice to avoid further shisha tobacco. Serial chest radiographs were performed at intervals of two weeks, six weeks, 12 weeks, and nine months. These demonstrated progressive resolution of the pulmonary opacities, with a clear chest radiograph at 12 weeks (Fig. 3). A spirometry performed at nine months was normal.

The impression was of bilateral pulmonary granulomatous nodules most likely related to shisha smoking, with complete resolution following cessation.

## Discussion

Shisha, hookah, and narghile are all forms of water pipes that have been used in Africa and Asia for smoking tobacco for nearly 400 years 1. They were developed by a 16th‐century physician with the intent of purifying the smoke via water. Over 100 million people worldwide regularly smoke shisha tobacco 2. Shisha has gained popularity in recent years in developed countries, particularly amongst young adults. Yet, the harmful health effects of shisha are not well known.

Many still believe that shisha smoke is safer because of the passage of the smoke through water; however, several studies have demonstrated a significant increase in lung cancer risk in shisha smokers 3. A study of young Saudi adults found that shisha smokers had a significant decrease in lung function parameters (Forced expiratory volume in one second (FEV1) and FEV_1_/Forced vital capacity (FVC) ratio) 4. According to one meta‐analysis, one session of shisha smoking exposes a user to a smoke volume of 74.1 L, compared to 0.6 L for one cigarette, and higher levels of tobacco toxins like nicotine, tar, and carbon monoxide 5.

Aside from the cancer and chronic obstructive pulmonary disease risk, there is the potential for increased risk of pulmonary infections as a result of shisha use as various parts of the device can act as a reservoir for pathogens. Alaidarous et al. obtained cultures from the water reservoir and mouth pieces from 10 different water pipe cafes and found a high frequency of bacterial contamination with resistant bacteria 6. A recent study showed increased risk of inhaling spore‐producing fungi, which were isolated from the inner parts of shishas 7. Aspergillus has been isolated from the water chamber of a shisha pipe in a leukaemia patient who developed invasive pulmonary aspergillosis 8. Mycobacterium tuberculosis has also been found to grow and survive inside shishas 9. The potential for transmission has been demonstrated in a cluster of pulmonary tuberculosis cases linked to the sharing of a water pipe in Queensland 10.

There have also been numerous case reports of acute eosinophilic pneumonia in shisha smokers. Dyal et al. described a 26‐year‐old female who had recently started shisha smoking, who was admitted with cough, dyspnoea, and pleuritic chest pain 11. Initial CT of the chest demonstrated small nodular opacities in the right lower lobe; however, the patient deteriorated, requiring admission to the intensive care unit where a repeat chest radiograph showed extensive bilateral pulmonary opacities. Bronchoalveolar lavage indicated 61% eosinophils. She was diagnosed with acute eosinophilic pneumonia and improved on prednisone. Several other cases following a similar clinical course have been reported, all affecting young adults 12, 13, 14. In all reported cases, the patients rapidly progressed to diffuse pulmonary infiltrates and were diagnosed on bronchoalveolar lavage with >50% eosinophils. In two cases, there was progressive respiratory failure leading to intubation and one that went on to need extracorporeal membrane oxygenation (ECMO). None of these patients had lung biopsies and histological assessment of the lung lesions.

There is only one other reported case of biopsy‐proven granulomatous inflammation as a result of Shisha smoking 15. A 20‐year‐old shisha smoker presented with similar symptoms to our patient and was found to have bibasilar lung nodules on CT. Video‐assisted thoracoscopic lung biopsy demonstrated necrotizing granulomas. He was treated with a course of prednisone, and a follow‐up CT scan at four months showed complete resolution of changes.

In our case, we were able to demonstrate necrotizing granulomas on biopsy with complete radiographic resolution of the lesions following cessation of shisha smoking and without specific therapy. Other causes of granulomatous inflammation, such as malignancy, vasculitides, foreign body, LCH, and hypersensitivity pneumonitis, were excluded clinically and on thorough histopathologic examination. A possibility of mycobacterial and fungal infection was considered and excluded with negative microbiological cultures of tissue biopsy specimen for fungi, a negative TB PCR, and cultures for both typical and atypical mycobacteria. Further testing to identify infection (e.g. 16s ribosomal RNA sequencing, fungal serology) was not pursued given the patient’s clinical improvement without antimicrobial therapy.

Sarcoidosis is also associated with granulomatous inflammation. However, the temporal profile of events, histological appearances of necrotizing (rather than non‐necrotizing) granulomas, and spontaneous improvement over a short period without the use of steroids would make sarcoidosis a less likely diagnosis.

In conclusion, we believe this was a rare case of necrotizing granulomatous lung disease linked to the regular smoking of shisha pipes. The case adds to the literature suggesting an unrecognized harm associated with shisha smoking and also emphasizes the need to consider an appropriate smoking history.

### Disclosure Statement

Appropriate written informed consent was obtained for publication of this case report and accompanying images.
